# If Teaching Is a Female Dominated Profession, Why Are So Few Leading the Profession?

**DOI:** 10.1177/19427751221137926

**Published:** 2022-12-02

**Authors:** Samira ElAtia, Leticia Nadler Gomez, Elissa Corsi

**Affiliations:** 1 University of Alberta, Canada

**Keywords:** Alberta, women leaders, education, challenging cultural and social norms, mentoring

## Abstract

Although the teaching profession is dominated by women in Canada, they are still under-represented in the top leadership roles in the education system. This study highlights the current situation in Alberta, examines the barriers to women progressing to the top positions in the field of education and presents the most recurrent suggestions as found in the literature. Such suggestions include, organizations taking an intersectional approach to recruitment, training, pay, and promotions; organizations recognizing the importance of healthy career-life balance for its teachers; existing leaders applying transformational leadership to help new and prospective leaders; and institutions establishing mentoring programs.

## Introduction

The increase of women in academia and other leadership careers has allowed for a greater exploration into the role of gender and leadership (Acker, 2014; Grogan & Shakeshaft, 2011 ; Huppatz et al. (2019); Ollilainen (2019); Ollilainen and Solomon (2014); Connell et al., 2015; Sperandio, 2015; Wyland, 2016). Although the number of women in educational leadership continues to rise, they are still under-represented in the top leadership roles (Hoff & Mitchell, 2008; Hoff et al., 2006; Kellar, 2013; Sanchez & Thornton, 2010; Smith, 2011; Tucker & Fushell, 2014; Wallace, 2002, 2004; Wallace & Wallin, 2015). Particularly, Wallace and Wallin (2015) document the journeys of women leaders which are filled with obstacles to obtaining a school superintendent’s position. Hence, the question arises, if the teaching profession is highly feminized, why are there so few women at the top?

The under-representation of women in various school and educational leadership roles in Alberta (and across the world) is concerning for two main reasons: First “[it] convey[s] to pupils a vision of society where it is legitimate that men occupy the most valued positions” (Moreau et al., 2007, p. 250). Second, the education system is “an institution through which gendered divisions are reproduced” (Hutchings, 2002 , p. 125, as cited in Moreau et al., 2007, p. 250). Therefore, there is an urgent need to recognize “the lack of cultural, racial or gender diversity in leadership and the dominant whiteness and maleness of educational leaders” (Blackmore, 2013, p. 147), as schools “constitute a key site in which democratic citizenship is understood and practiced” (Blackmore, 2013, p. 148).

In democratic societies of the 21st century, as Blackmore (2013) stated, “it can no longer be argued that women lack the necessary skills or ambition or that the pool of qualified women does not exist. The question therefore becomes whether organizations recognize the escalating expectations of leaders together with the intensification of educational labor pitted against the demands of managing family/work conflict” (p. 151).

In the province of Alberta, Canada, the percentage of women in leadership roles, as defined by both the School Leadership Quality Standard and the Superintendent Leadership Quality Standard (LQS and SLQS), declines as the level of leadership increases. The Alberta Teachers Association (ATA) has never had a female Executive Secretary, and a mere 13% of the province’s Superintendents are women. Yet, the Alberta Teachers’ Retirement Fund (ATRF) reported in 2017 that 76% of the teaching population was female. Despite this discrepancy, there has been little formal study in this area. Data from the ATRF (2017) show a troubling trend:

– In 2006, 83% of the teaching profession was comprised of women in their 30s.
– In 2015, this number of women in their 30’s significantly decreased to 70%.

Women between the ages of 35 and 39 are leaving the profession just as they would be expected to be moving into leadership positions. This begs the question, why are these women leaving and why are they not ascending the professional ladder? More troubling than this are the results obtained from the *Women in Educational Leadership Needs Assessment Survey*, conducted by the ATA in 2018/2019, (ATA Research, 2020). Participants of this survey identified multiple barriers to leadership, with some recurring ones, such as the impact of family leave and unpaid labor; globally, women and girls are responsible for 75% of unpaid care and domestic work. This includes caring for children, older parents, and relatives. Similarly, research at the intersection of gender highlights various factors that come to play for women in leadership: Family life, caretaking, socio-cultural roles and perceptions, and economics. The women in this study reflected much of what is stated in research about women in leadership in general. Reasons for the under-represented of women in the upper levels of administration may be competing urgencies of family life, gender bias, and/or stereotypical perceptions and expectations (Seyfried & Diamantes, 2005). Researchers also reported that “the workforce is patriarchal in design and that the ideal worker is [deemed to be] male” (Chami, 2016, p. 97), and “gender imbalance is inhibited by institutionalize gender systems, social practices and structures often seen in education organizations” (Kairys, 2018, p. 932).

In Alberta, the under-representation of women in school superintendency has, thus far, caught the attention of the ATA. In 2017, Shelley Magnusson, ATA Executive Staff Officer, published an article, *Viewpoints: Open the door to gender equity*, in the ATA magazine, in which she stated the “need to take thoughtful and deliberate action to increase the number of women in leadership positions” (Magnusson, 2017, para. 2). This same issue was highlighted in the above mentioned survey published by ATA Research (2020). As the pandemic brought the struggle of working mothers to the forefront of the public’s attention, as well as women in general, who make up most of the teaching workforce, and the lack of literature reporting on gender imbalance in educational leadership in Alberta, the need was identified to conduct a research project in partnership with a diverse base of experts that covered both theoretical and practical specializations. This included, as the core partnerships, the University of Alberta (UofA), the ATA, and the College of Alberta School Superintendents (CASS). This article marks the start of a 3-year research project and draws from the academic literature as well as the gray literature, published mainly in North America.

## Objective of Current Research

The main aim of this work review was to provide a clear picture of the under-representation of women in educational leadership using the most recent academic work published on the matter. This review is the first step in a wider research project, for which we plan to investigate the lived experiences of women in educational leadership in Alberta to establish baseline data regarding the participation of women in educational leadership. At a later stage, the project will also entail the need to co-create with our partners policies and systematic administrative procedures that will remove barriers to career progression for women in educational leadership. We also intend to develop a data acquisition model that will guide decision-making and allow stakeholders in Alberta to continue to document the gains of women in educational leadership.

Building on this inquiry, we also intend to examine the potential reasons why women are seemingly excluded from the top positions. Is this because of the previously mentioned barriers in relation to gender bias, stereotyping, expectations, family commitments, or a combination of these; or, perhaps, there are other forms of discrimination involved, that is, intersectional identities are being applied? The concept of “intersectionality refers to the way in which multiple forms of discrimination—based on gender, race, sexuality, disability, and class, etc.—overlap and interact with one another to shape how different individuals and groups experience discrimination” (Gender and Development Network [GADN], 2019).

## Methodology of the Literature Review

For this article, we conducted a systematic review within a specific period to develop a clear and comprehensive overview of the underrepresentation of women in educational leadership. To conduct this review, we used both the EBSCO and ERIC databases available at the University of Alberta library and then Google Scholar and key websites. We followed four main steps: (1) Identifying keywords (see Table 1) in relation to our main research questions (2) selecting the relevant studies based on strict search limiters (see Table 1) (3) identifying the challenges, their causes and the recommendations to overcome these challenges (see Table 2 (4) synthesizing the content according to the most recurrent themes emerging from both the academic and the gray literature. Overall, we examined peer-reviewed articles, theses, dissertations, and professional literature (i.e., websites and reports) that we organized by keywords in an Excel document, in which we recorded the title of each source, name of authors, publication date, as well as key paragraphs of each article. We highlighted relevant citations, using a systematic, color-coding method and did so for three topics: the challenges; the causes of the challenges and the recommendations to overcome these challenges.

**Table 1.** Keys Words & Search Limiters.^1^

Intersections of gender and women in school leadership	2017–2022 scholarly peer-reviewed journals (North America only):
	Academic journals (498)
	English (412)
	Many of the 412 articles appeared several times. In the end, there were 275 different articles. We reviewed the titles of each one of them and selected the most relevant articles (30).
The ideal worker and maternity/parental leave	2017–2022 scholarly peer-reviewed journals (North America and English only):
	Academic Journals (548)
	We reviewed the titles of each one of them and selected the most relevant articles (11).
Gender stereotyping and educational leadership and educational administration	2017–2,022 scholarly peer-reviewed journals (North America and English only):
	Academic Journals (13)
	We reviewed the titles of each one of them and selected the most relevant articles (5).
Mentorship and women and educational leadership	2000–2022 scholarly peer-reviewed journals (North America and English only):
	Academic Journals (96)
	We reviewed the titles of each one of them and selected the most relevant articles (16).

**Table 2.** Keys Themes.

Challenges	Gender and race
	Maternity/Parental Leave
Causes of the challenges	Gendered organizations
	Hiring process
	Covid-19 pandemic
Recommendations	Intersectionality approach in educational leadership research
	Career-life balance
	Agency of current leaders
	Mentoring program

Dr. Tanya De Mello^2^ stated in her doctoral research that women and racialized people are under-represented in professional roles in all industries across Canada (De Mello, 2020). The education system is no different. For instance, visible minorities from Black (Brooks & Jean-Marie, 2007; Elonga Mboyo, 2019) and Asian descendants (Liang & Peters-Hawkins, 2017; Liang et al., 2018) are rarely making it to the top of the education system, yet these minority groups are significant and present in the Canadian (and Albertan) landscape.

## Women’s Leadership Versus the Barrier of Intersectional Gender and Race

Women are not a homogenous group and minority women, in particular, experience systemic oppression, including in the field of education. Research has shown that “gender is intermeshed with other aspects of social life” (Ramazanoğlu & Holland, 2002, p. 163) forming unique modes of discrimination and systemic oppression in the workplace (Macias & Stephens, 2019). Generally, “minorities are vastly underrepresented in every position [. . .], particularly leadership” (Ramazanoğlu & Holland, 2002, p. 169), including in the education system. Fuller et al. (2019) found that racial/ethnic and gender attributes influenced the odds of visible minority group educators obtaining leadership positions. In the US, research on the intersection of gender and race shows a gap between non-racialized and racialized women, especially among Black women in senior-level educational leadership positions (Aaron, 2020; Johnson, 2021). More specifically, “women and men of color still have lower odds of becoming a principal relative to white men despite the relative rapid diversification of assistant principals” (Fuller et al., 2019, p. 147).

Zajicek et al. (2020), emphasized the relevance of using an intersectional perspective to explore gender patterns in specific occupations and within racialized groups. Crenshaw (2018) also insisted on placing the marginalized at the center which is “the most effective way to resist efforts to compartmentalize experiences and undermine potential collective action” (2018, p. 167). Intersectionality must be used as the lens through which to better observe and understand the complicated factors and ideologies at play within the experiences of women in educational leadership; dependant on time, space, and lived reality. Intersections of gender must be used to support understanding around this topic, using the unique experiences of each woman as a basis for achieving equity, diversity, and inclusion. Ultimately, as Rosemary Brown (the politician, activist, and Canada’s first Black female member of a provincial legislature) said, “We must open the doors and we must see to it they remain open, so that others can pass through. Until all of us have made it, none of us have made it” (Women of Influence, 2016).

Despite such inspirational and wise words as those of Brown, it seems that society still has a long way to go. Both in the corporate and non-corporate world, intersectionality in the form of gender discrimination can exist on more than one level, making the obstacles even harder to overcome for women, though, for some, this does not stop them from trying.

### Women Labeled as “Non-Ideal Workers” in Gendered Organizations

Women are not passive agents when it comes to their career path progression (Smith, 2011), and they work hard at navigating professional ambition with family commitments. Female teachers often take on additional training and certification to become educational leaders, but they do not necessary obtain those positions (Allred et al., 2017). Generally, in organizations, being persistently gendered (Acker, 1990), women encounter roadblocks in the workplace. The dominant cultural norm implicitly associates women with family responsibilities and portrays them as a *non-ideal worker*. Symbolically, “gender, sexuality, and bodies can be thought of as [problematic] organizational resources” (Shafritz et al., 2016, p. 422) and are often used as grounds for exclusion or objectification. As a result, women experience a double form of discrimination in comparison to men. They first experience discrimination through their physical and physiological bodily “constraints,” and then through the higher level of control exercised (directly and indirectly) by the managerial body on those “constraints.” The exercise of this control is reflected in organizational substructures.

Unlike for men, for women, organizational substructures impact women’s daily workspace and extend to their living spaces, bodies, and physiological functions. In this sense, an abstract job carried out by a disembodied individual working in a neutral organizational substructure is, in fact, not so much neutral but male by default. Substructures are never gender-neutral; they are clearly gendered at the expense of women. In other words, there are two realities—understandings and meanings—these co-exist, persist, and reproduce in organizations. This form of duality is embedded in the organizational processes and can be reflected in various activities, including the production of texts and job evaluations, to name a few (Shafritz et al., 2016).

Organizational practices produce gender divisions that are reflected symbolically in power distribution and dynamics based on job fit perceptions. Thus, both gender and sexuality contribute to the production and reproduction of gendered hierarchies. Beyond symbols, images, and forms of consciousness, daily interactions between individuals also contribute to reproducing gendered organizations. Those interactions are shaped by the internal mental models of individuals, which are developed by their own understandings and construct of the gendered structures of work and their implicit demands for gender-appropriate ways of being, thinking, and acting (Shafritz et al., 2016). The danger in “framing the ideal worker norm and flexibility bias as a mother’s issue may help perpetuate gender inequality by limiting the scope to which these norms are theorized and empirically examined” (O’Connor & Cech, 2018, p. 823).

For serious change to occur, family responsibilities should be perceived as essential and apart from work responsibilities, this being immune from the former; and those at higher organizational levels would need to be willing to challenge the concept of an *ideal worker* (Borgkvist et al., 2021). However, female stereotypes and the expectation of greater family commitments and demands being more prevalent for women still seem to make women less attractive to employers and more of a liability than an asset. In other words, as long as the perception of women is associated with motherhood, chances are that maternity leaves add another layer of difficulty to working women, especially for those wanting to climb the leadership ladder. This is a topic that is at the heart of our research project and that we see the need to explore further. In the next section, the outcome of an investigation of the relevant literature on this issue is presented, specifically, regarding the role of maternity leave in women’s career paths and destinations and, by extension, the role of parental and family leave.

## Maternity/Parental Leave

In addition to the barriers toward female workers, such as gender discrimination and family responsibilities (Wyland, 2016), there were very few studies found of research conducted in North America on the connection between women’s career path progression and maternity, parental, and/or family leave. In this article, we refer to these terms as defined by the ATA. For the ATA, the purpose of *maternity leave* is time away from work to “recognize the medical needs of a mother who gives birth”; this is only available to birth mothers and is the equivalent of up to 16 weeks off work. It is possible to extend maternity leave to up to 18 months. It is then called *parental leave* and is available to “birth mother, partner, or adoptive parent [. . .], which is a minimum of 37 weeks and can be shared by both parents” (Alberta Teachers Association [ATA], 2022, para. 6). Teachers may “be entitled to more leave than is mandated by law” and may they be able to extend it by taking “a general leave of absence or childcare leave of absence for up to one year after the first year of the baby’s life,” (para. 18) all of which depends on the collective bargaining agreement under which the teacher is working. Although the terms maternity leave and parental leave are different and cannot be used interchangeably, for the purpose of this article we see them as one particular type of leave in relation to children; thus, we herein refer to them as maternity/parental leave as available to all eligible Alberta teachers when appropriate (ATA, 2022).

The lack of research in the area of maternity/parental leave is concerning as “the effects of parental leave policies are numerous and extensive due, in part, to the fact [that] the interpretation of parental leave policies affects both people’s professional and personal lives” (Martin, 2018, p. 113). According to Maxwell et al. (2019), regarding maternity leave, “the real impact is that they [organizations] effectively require women to work harder to keep pace within a linear, competitive career track and to strategize for career advancement.”

After World War I, feminists and female trade unionists pushed for “fair labour standards for working women—including paid maternity leave—as a matter of social justice and international security”(Siegel, 2019). As a result, in November 1919, the “International Labo[u]r Organization adopted the Maternity Protection Convention of 1919 (Siegel, 2019). In Canada, leave from work for new mothers started when “BC introduced the Maternity Protection Act of 1921. This legislation enabled women to take a limited leave of absence before and after giving birth and made it unlawful to dismiss women for these absences” (Legislative Assembly: Province of British Columbia, 2015, paragraph 9). The establishment of a maternity leave program, in Canada, was one of the 167 recommendations outlined by the Royal Commission on the Status of Women in Canada (established in 1967) to the federal government (Morris, 2006). At the beginning of World War II, in August 1940, the Employment Insurance Act was introduced in Canada; however, this did not address the need for permitted maternity leave.

“Maternity leave, as we currently understand it, was first introduced in BC in 1966. Five years later, the federal government followed suit, amending the Canada Labour Code” (Morris, 2006). It then became a significant topic of advocacy for labor unions leading to further gain for the female labor workforce and beyond (adoption leave, paternity leave, and parental leave) (Morris, 2006). In North America today, parental leave can vary between 1 and 3 years, (Chami, 2016) but despite significant gains over the last few decades in this area, inequalities in the workplace persist for women. Additionally, policies supporting the extension of maternity leave seemed to “reinforce the message that women belong at home raising babies” (Chami, 2016, p. 100). In other words, maternity/parental/family leave is a complex matter for female teachers and may negatively affect the progression of their career paths.

In European countries, there are various different models of parental leave (Dobrotić & Blum, 2020). While a few do not have paid parental leave at all, across the majority of Europe, more and more fathers are taking parental (paternal) leave; however, the division between women and men remains asymmetrical. Taking parental leave for both mothers and fathers who hold stable positions is perceived as an act that slows down their career advancement. In addition, Samtleben et al. (2019) showed that supervisors often support traditional gender norms. They also discovered that parents are more likely to extend their parental leave if the employer finds a substitute; this is less likely to happen for male workers. Staffing strategies of male workers, fear of career penalties, and gendered expectations from employers/supervisors, rather than family-friendly workplace policies, have greater impact on the length of leave as opposed to career progression (Samtleben et al., 2019). For female workers, things are different: the presence of traditional gender norms in the workplace enable mothers to take longer periods of parental leave; however, then they also experience detrimental effects regarding their career advancement (Samtleben et al., 2019). In short, in organizations that support traditional gender or ideal worker norms, parental leave has greater negative career consequences for mothers than for fathers. Women also suffer from others’ perceptions of them being less competent, less focused on work, and expected to stay home to take care of their young children (Samtleben et al., 2019). The women who attempt to break free from these cultural norms and expectations can still be faced with barriers.

## Women Struggling to Take the Next Step Up the Career Ladder

Although the teaching profession is highly feminized worldwide (Bush, 2021), female teachers, just like their male counterparts, and to which they express through additional training or mentoring, aspire to obtain school and district leadership positions (Murakami & Törnsen, 2017). In leading schools and districts, female education professionals do not fit into cultural norms nor traditional social roles and expectations. In the workplace, the very few women who make it to the top are confronted with gender biases (Murakami & Törnsen, 2017), which can also affect them during the application and interview processes.

Women and men portray both similarities and differences on written applications and at interviews for educational leadership positions (Finch et al., 2019). In terms of language used in school leadership contexts, Finch et al. (2019) found that, on the one hand, “both women and men write about their beliefs for education and schooling and discuss leadership responsibilities. [. . .] They both write about previous professional experiences” (p. 319). On the other hand, “women are more likely than men to write about learning and professional development and for having expertise in curriculum and instruction in order to provide leadership in this area. [. . .] women emphasize collaboration and building relationships with the school community. In contrast, men are more focused on outcomes and successes likely to result from their leadership” (p. 320). However, it is not clear if it is these differences in the use of language, or other factors (perhaps gender bias) that contribute to a hiring committee selecting a man over a woman.

Fuller et al. (2019) concluded that “personal characteristics influence the odds of obtaining employment” (p. 148), and women superintendent candidates feel that school boards continue to prefer men over women for these higher positions (Sampson & Davenport, 2010). Some women, however, disagree with this; thus, researchers have suggested that school boards, as with all other public sector employers, must comply with equality principles in terms of hiring (Blackmore, 2013, 149), and change certain aspects of their recruiting process to ensure fairness and equity (Fuller et al., 2019 , p. 59). Moreover, Newton (2006) suggested that employers “avoid recruitment practices perpetuating the notion that the superintendent’s position is ‘male’ . . . [and] encourage more women to apply for position vacancies” (p. 26). Over the years, mentoring programs have become a common means of building a pool of potential leaders.

## Mentoring: A Potential Remedy to Help Women Win Top Educational Leadership Roles?

To overcome the under-representation of women, particularly racialized women, Johnson (2021) outlined “the need for more mentors, safe spaces, and pathways to leadership for Black women in education” (Johnson, 2021, p. 637). Mentoring and additional resources can be beneficial to women’s professional and personal lives and aid in confronting intersectional stereotypes (Aaron, 2020; Bynum, 2015; Jernigan et al., 2020). Thus, Santamaría and Jaramillo (2014) emphasized the importance of informal mentors as well as any other forms of teachings received. Both formal and informal mentorship can be beneficial to women aspiring to become school superintendents (Copeland & Calhoun, 2014; Howard et al., 2017; Kamler, 2006; Searby & Tripses, 2006) as well as personal connections (Jernigan et al., 2020). In sum, institutions need to give greater importance and support to mentoring programs (Block & Tietjen-Smith, 2016).

While mentoring programs are key to helping women break the glass ceiling, ultimately, they rely solely on a woman’s individual agency to obtain equal opportunities in the workplace and a fair division of labor in the household, as suggested by Kiraly and Tyler (2015). This, however, is not just insufficient, it is contradictory (Eisenstein, 2010). Systemic barriers and chronic social problems need to be addressed at the systemic level through the lens of system thinking (Coulson et al., 2012; Mayer & Le Bourdais, 2019; Shepherd, 2017; Stroh, 2015); focusing on mentoring only may hinder the root of the problem.

As mentioned in the beginning of this article, the Covid-19 pandemic has exarcerbated systemic barriers in multiple fields of employment, including education, and made the life of working mothers more challenging than before. If these women were involved in some mentoring programs before the pandemic hit, chances are, they had no other choice but to drop out to adjust to the new demands. Indeed, many of these women were required to take on the additional responsibility of their own children’s education and move themselves, their careers, and their leadership ambitions down their list of priorities.

## Women in Educational Leadership in the Light of the Covid-19 Pandemic

In the Covid-19 era, the role of women is rapidly changing; responsibilities are increasing and inequalities are becoming more pronounced and prevalent in today’s society. With schools closed, the unpaid work of women has increased and the fragility of the participation of women in the workplace (especially in the teaching profession) has been brought to the forefront. The economic downturn has been dubbed a “she-cession” (LeanIn, 2020; Lewis, 2020; Power, 2020), and the impact on the working lives of women is beginning to be documented (Chung, 2020; Promundo, 2020; United Nations, 2020; Wenham et al., 2020). Women during this time have had to wear many hats – mothers, teachers, caregivers, housekeepers—and as the pandemic carries on, some are having to leave their careers to focus on other responsibilities. Economic gains that have been accumulated over the years are being wiped out in months and we are in a “gender-regressive” pandemic (Power, 2020, p. 68).

Women are entering a “third shift” (Chung, 2020; Power, 2020), exacerbated by the pandemic; the undervalued and unpaid emotional labor of women. School leaders are impacted greatly by this third shift because they are not only responsible for the emotional well-being of their own family members, but also that of their, staff, students, and, sometimes, even the families of their students. Basically, school leaders are responsible for making sure that everyone is alright.

Within educational leadership there are struggles around power and the complexity associated with feminist thought (Blackmore, 2013). It is essential to explore the issues of family leave and the unpaid work of women within a wider context of social relations, including these relations of power. Intersectionality allows for researchers to examine interwoven educational injustices and highlight relationships between gender and society (Agosto & Roland, 2018; Cho et al., 2013; Collins & Bilge, 2016; Natapoff, 1995; Scanlan & Theoharis, 2016).

There are many different interpretations, definitions, and practices of the theories around feminism, and, as stated by Wallace and Wallin (2015), “one of the first learnings that must be understood is that *there is no* ***one*** *understanding of feminism* that is, or should be, ‘applied’ to educational administration” (p. 81, emphasis in text). Other authors, such as Tong (2014) provided an analytical frame for feminism through the exploration of seven different overarching groupings: Liberal feminism, radical feminism, Marxist and socialist feminism, psychoanalytic and care-focused feminism, existentialist and postmodern feminism, women of color feminism, and, most recently, ecofeminism. However, the one strand that unites feminism is the commonality contained in the experiences of women in a patriarchal world within a masculine culture (Crotty, 1998; Wallace & Wallin, 2015), such as those of female teachers looking to advance in the education system.

Thus, addressing the under-representation of women in school superintendency with a feminist approach means changing the status quo and, so, also addressing the over-representation of white men. It means dismantling, “nation-building practices [that] have advanced the social and economic power of white settlers [. . .] [with] racist, sexist, and homophobic colonial practices” (McLean’s, 2018 , p. 33). In that sense, this links the possibility of accessing top jobs not to women’s individual agency but to over a 100 years of white male colonialism, and of persisting white ignorance that leaves very little room to minority groups (Mills, 2007 ). Yet, the academic literature in not as radical in terms of suggestions to counter the under-representation of women in the top roles of the education system.

## Overcoming the Under-representation of Women in Educational Leadership: What the Literature Suggests

To increase women’s representation in educational leadership positions, the academic literature highlights various important aspects: (1) Using an intersectionality approach to academic research and professional praxis; (2) Restructuring workplaces to offer new work models that enable leaders to reconcile career progression and work-life balance; (3) The agency of current leaders to change the status quo; and (4) Mentoring programs.

### Intersectionality

Research at the intersections of race and gender in the field of educational leadership has the potential to offer critical analysis and insight on women’s career paths and progress (Chase & Martin, 2021); thus, this is becoming a popular approach in studying women and educational leadership (Odell, 2021). Looking at different aspects of female teachers’ career such as the hiring process and the mentoring programs offered to them can provide a greater understanding of the challenges they encounter. In other words, an intersectionality approach can help anticipate and challenge potential discrimination and ensure equality and equity for all teachers and future educational leaders (Macias & Stephens, 2019).

### Career-Life Balance

The happiness and well-being of teachers, superintendents, and other school administrators should be considered to make “a positive contribution to the success of the organization” (Keller, 1999 , as cited in Seyfried & Diamantes, 2005, p. 67); thus, an appropriate career-life balance is deemed vital. Moreover, Bascia and Young (2001) , as cited in Seyfried & Diamantes, 2005) recognized “the powerful [and beneficial] effects of home and family circumstance on [one’s] career,” which are relevant to “both men and women as they fulfill their equally important [familial] roles” (p. 67). However, an article by Moodly and Toni (2017), emphasized that “the working environment encroaches more and more on family life and family support often perpetuates the acceptance of worktime eating into family time” (p. 151). Teachers shouldn’t have to “sacrifice family time in order to ensure a successful career of either parent” (p. 151). In response to this, it is essential that school districts revise “their organisational values, policies, and procedures and introduce critical changes to accommodate the needs of non-traditional leaders (women, racial minorities, [working mothers and fathers,] and the much-younger/older” (Liang et al., 2018, p. 637).

### The Agency of Current Leaders

Leaders (both men and women) need to apply themselves to “stimulating re-thinking around policy-planning and implementation” and disrupt “traditionally accepted processes” to ensure that women are “represented at the highest levels of leadership” in education (Moodly, 2021, p. 201). In addition to this, men in leadership roles could take it upon themselves to exercise their agency, disrupt inequalities, and challenge “institutional cultures that continue to inhibit the advancement of women towards leadership” (Moodly, 2021, p. 202). To facilitate this, gender inequality and intersectionality need to be part of the discourse around policy and institutional structure, and the “normative assumptions on which policies are constructed” must be challenged (Moodly, 2021, p. 201). Furthermore, lifecycle phases, with regard to work, life, and family life, are often not in linear order; thus, policies need to be “adaptable to the individual’s growth path,” whether they be male or female (Moodly & Toni, 2017, p. 151).

### Mentoring Programs

Learning from other’s experiences, regarding both individuals seeking educational learnership roles and organizations becoming more gender neutral and inclusive in leadership positions available, would benefit all those involved. With this in mind, the ministry of education, schools, universities, colleges, and professional organizations “could collaborate and establish formal mentoring programs with specific structures for issues related to females” (Connell et al., 2015, p. 53). Suitable and effective mentors could facilitate prospective women applicants for leadership roles in the education system, and women who already hold these positions, “to provide [them with] connections, opportunities to expand their knowledge and skills, encouragement, and feedback on [their] performance” (p. 53).

At the university level, undergraduate and graduate programs could all provide career planning to show women that higher level jobs are open to them to give them an achievable goal to reach and to stimulate their ambitions (Connell et al., 2015). Another way of advocating increased inclusion of women in top leadership roles is by holding “forums and other opportunities for students to interact with females in influential leadership positions could encourage potential future leaders in education, support them in developing their skills, and remove some of the barriers to females in leadership” (p. 53). Further, “institutions could provide students [with] explicit information on career paths, and knowledge about gender bias in the workplace” (p. 53).

At the organization level, the content of superintendent preparation programs should align with the four components of Bass’ theory of transformational leadership: Individual consideration, intellectual stimulation, inspirational motivation (charismatic leadership), and idealized influence (Howard et al., 2017, p. 68). This will empower those entering leadership positions and enable their followers to gain the most from their experiences.

## Discussion

The purpose of this review was to identify the most recurrent challenges outlined in the literature in relation to the underrepresentation of women in educational leadership with a special focus on school superintendents to gain further understanding of the current debate. This review is part of a bigger project for which we are currently collecting data throughout the entire province of Alberta, and only future publications drawing from the data collected will allow us to bring insights that will push readers to reconsider the practices of organizations when considering promotion and retention of women in executive leadership in schools and districts. In short, we see the necessity to move beyond individual explanations for why women might be underrepresented in the upper management of educational organizations, but it is simply too premature to address it in this article.

## Conclusion

Although the number of women in the teaching profession is high, the numbers of women leaders in education continues to rise, they are still under-represented in the top leadership roles. This under-representation of women in top leadership positions is a significant issue in the educational system in Alberta and in many countries around the world. The causes of this under-representation were investigated through a systematic literature review, and recommendations based on the findings have been presented.

In addition to gender discrimination, aligned with cultural norms, traditional social roles, and expectations, racial/ethnic attributes also inhibit from obtaining leadership positions. The intersection of gender and race means that it is even harder for women to achieve success to the higher levels, to the extent that some women do not even attempt to do this.

Women being labeled as non-ideal workers by institutions caused barriers to women as some organizations saw them as unreliable liabilities that were not always competent and unable to focus on their work as family responsibilities took priority. This extended to problems with the financial burden and the loss of work hours due to maternity/parental/family leave, which restricted women’s passage up the career ladder, more so than that of men. These problems were exacerbated with the onset of the Covid-19 pandemic and the restrictions that came with this.

Certain recommendations were made to rise above these aforementioned challenged blocking the progression of women in educational leadership. These included organizations taking an intersectional approach with regard to recruitment, training, pay, and promotions. Also, organizations must recognize the importance and actively try to achieve a healthy career-life balance for its teachers, regardless of their gender and position in the educational hierarchy. Existing leaders could and should help new and prospective leaders at all levels; this is best achieved through mentoring programs. By applying transformational leadership and conveying narratives of both positive and negative experiences to mentees, mentors can help “re-define the territory [. . .] for the young women who will lead soon” (Grogan, 2010, p. 786).

Only in supporting women through their journey in educational leadership and shifting the paradigm pertaining to family leave and unpaid work will women achieve gender parity. As Virginia Woolf noted, “The obstacles against her [(women)] are still immensely powerful—and yet they are very difficult to define” (Woolf, 1931), but these obstacles must be acknowledged by men, women, and organizations for them to be addressed, challenged, and eventually overcome. After all, as Crenshaw (2021) said “lifetime achievement [and by extension, career progression] is not a singular or individual narrative, but a reflection of history and collective action, of timing and opportunity” (p. 1709).

## Notes

1. We must acknowledge some limitations regarding our search parameters. Focusing on recent publications led to missing the work of Dr. Judy A. Alston—and probably others—an important scholar whose research interest from 2000 to 2012 was prominently on Black women in educational leadership, including female school superintendents. We are conscious that through this publication we are producing knowledge, and who we cite and do not cite contributes to shaping the academic and mainstream knowledge. Therefore, if the readers find that some scholars were omitted, it is strictly related to our search limiters. In future publications, we will reflect further on what search limiters to use to be more inclusive as knowledge production is not neutral, it is political.

2. Dr. Tanya De Mello is a human rights lawyer and activist, consultant on diversity, equity and inclusion, and frequent guest speaker for the ATA.
